# Variable selection in social-environmental data: sparse regression and tree ensemble machine learning approaches

**DOI:** 10.1186/s12874-020-01183-9

**Published:** 2020-12-10

**Authors:** Elizabeth Handorf, Yinuo Yin, Michael Slifker, Shannon Lynch

**Affiliations:** 1 Biostatistics and Bioinformatics Facility, Fox Chase Cancer Center, Reimann 383, 333 Cottman Ave, Philadelphia, PA 19111 USA; 2 Cancer Prevention and Control, Fox Chase Cancer Center, Young Pavilion, 333 Cottman Ave, Philadelphia, PA 19111 USA

**Keywords:** Variable selection, Social environment

## Abstract

**Background:**

Social-environmental data obtained from the US Census is an important resource for understanding health disparities, but rarely is the full dataset utilized for analysis. A barrier to incorporating the full data is a lack of solid recommendations for variable selection, with researchers often hand-selecting a few variables. Thus, we evaluated the ability of empirical machine learning approaches to identify social-environmental factors having a true association with a health outcome.

**Methods:**

We compared several popular machine learning methods, including penalized regressions (e.g. lasso, elastic net), and tree ensemble methods. Via simulation, we assessed the methods’ ability to identify census variables truly associated with binary and continuous outcomes while minimizing false positive results (10 true associations, 1000 total variables). We applied the most promising method to the full census data (*p* = 14,663 variables) linked to prostate cancer registry data (*n* = 76,186 cases) to identify social-environmental factors associated with advanced prostate cancer.

**Results:**

In simulations, we found that elastic net identified many true-positive variables, while lasso provided good control of false positives. Using a combined measure of accuracy, hierarchical clustering based on Spearman’s correlation with sparse group lasso regression performed the best overall. Bayesian Adaptive Regression Trees outperformed other tree ensemble methods, but not the sparse group lasso. In the full dataset, the sparse group lasso successfully identified a subset of variables, three of which replicated earlier findings.

**Conclusions:**

This analysis demonstrated the potential of empirical machine learning approaches to identify a small subset of census variables having a true association with the outcome, and that replicate across empiric methods. Sparse clustered regression models performed best, as they identified many true positive variables while controlling false positive discoveries.

**Supplementary Information:**

The online version contains supplementary material available at 10.1186/s12874-020-01183-9.

## Background

The Precision Medicine Initiative suggests that environment, along with genes and lifestyle behaviors, should be considered for cancer treatment and prevention. Nevertheless, the impact of social environment, or the neighborhood in which a person lives, remains understudied [1]. Compared to the biological level where empirical, high-dimensional computing approaches, like genome-wide association studies (GWAS), are often used for hypothesis-generation, risk prediction, and variable selection, empirical methods are only beginning to be employed at the environmental level [2, 3].

Social environment, as defined by a patient’s neighborhood of residence, is particularly relevant to the study of cancer health disparities. Neighborhood boundaries can be defined by US Census tracts (smaller geographic areas than a county). These neighborhoods can be described by variables measuring economic (e.g., employment, income); physical (e.g., housing/transportation structure); and social (e.g., poverty, education) characteristics [4, 5]. Studies linking US Census data with state and national cancer registry data show that neighborhood can help explain differential cancer incidence and mortality rates beyond race/ethnicity or genetic ancestry, and that neighborhood environment often exerts independent effects on cancer outcomes [6, 7].

Methodological challenges have limited the incorporation of neighborhood data into Precision Medicine. Most studies use a priori variable selection approaches, but there are no standard variables to represent particular domains (e.g. poverty, education, employment, etc.), which has limited translation of social environmental variables into clinical use. In the few studies using empiric selection approaches, variable selection and replication of findings were complicated by the high degree of correlation among US Census variables. For instance, similar to a GWAS, we previously conducted a neighborhood-wide association study (NWAS) in both black and white men in Pennsylvania and agnostically identified 22 US census variables (out of over 14,000) significantly associated with advanced prostate cancer [3]. In the first NWAS, social support was identified as an important neighborhood domain, but 2 very similar variables were identified to represent this domain *(% male householders living alone* vs *%male householders over 65 living alone in a non-family household*). Thus, multicollinearity (the presence of many highly interrelated variables) is a challenge for variable selection and replication.

The systematic assessments offered by machine learning algorithms, which allow for high dimensionality and collinearity, may be useful for analyses of neighborhood data. In this manuscript, we broadly use the term “machine learning” to refer to any computational method which selects variables automatically, without direct input from a human analyst. While the main objective of machine learners is often predictive accuracy, an additional objective is variable selection and determining which features are truly important. This is analogous to the goals of variant discovery vs risk prediction in genetic studies [8, 9].

Motivated by the previous NWAS of prostate cancer cases in Pennsylvania, we sought to understand which machine learning algorithms are most effective for identification of neighborhood factors which have a true association with a health outcome. Machine learning algorithms are often judged by comparing predicted vs. observed outcomes in an independent test set. We cannot use this paradigm to evaluate variable selection, however, as the true underlying variables associated with a given outcome are unknown. This motivated use of a simulation study, where we generated outcomes that are dependent on a small subset of the potential predictor variables. We then applied several popular machine learning approaches, including lasso, elastic net, hierarchical clustering, and random forests, and assessed how well each method identified true positive variables while minimizing false negatives. We compared the results to traditional regression with correction for multiple testing. Finally, we applied the top performing machine learning approaches to our original NWAS dataset, and compared findings from these analyses to our first NWAS in white men.

## Methods

### Candidate methods for discovery of important variables

Below, we describe methods for variable selection where both *p*, the number of potential predictor variables, and *N*, the number of observations, are large, and discuss how these methods can be applied to analysis of neighborhood-level covariates. We identified methods which provide objective and automatic variable (feature) selection for both continuous and binary outcomes. We also limited our evaluation to methods with largely automated tuning, which are readily implemented using standard R packages, and which one can run within reasonable timeframes. Ultimately, the methods we identified fell within two broad categories: penalized models, and ensemble tree-based methods.

#### Standard regression models

The simplest approach to variable selection is similar to the GWAS and NWAS approach [3]. A series of univariable tests are conducted to determine the relationship between each possible predictor and the outcome. Variables which are statistically significant after correction for multiple-testing [10] (i.e. ‘top hits”) are then replicated in a separate set of samples [11]. Although this approach is simple and easy to implement, the separate regression models ignore any correlation structure between candidate predictors. This may lead to selection of a large number of highly correlated variables, necessitating further variable selection steps, as described in the NWAS manuscript [3]. We included this approach as a baseline for comparison, to demonstrate the degree of improvement more advanced methods can provide.

#### Sparse regression models

Penalized regression addresses some of the limitations of standard regression for high-dimensional data. A useful class of these models provide shrinkage which enforces sparsity; that is, many of the parameter estimates are shrunk to exactly zero [12]. Sparse models have several advantages over traditional regression, including reduced overfitting (which improves prediction), accommodating multicollinearity, and the ability to fit models where *p > n*. They can also be used for variable reduction, where a zero parameter estimate indicates that the variable is not an important predictor.

##### Lasso penalized regression

The Least Absolute Shrinkage and Selection Operator (lasso) includes a L1-norm (absolute value) penalty that shrinks many parameter estimates to exactly zero [12, 13]. Thus, variables with non-zero coefficients can be considered the important predictors for the outcome of interest.

For a linear regression, the lasso solution is found as

1 $$ \underset{\beta }{\min}\left\{\frac{1}{2N}{\sum}_{i=1}^N{\left({y}_i-{\sum}_{j=1}^p{x}_{ij}{\beta}_j\right)}^2+\lambda {\sum}_{j=1}^p\left|{\beta}_j\right|\right\}, $$

where *N* is the number of observations, *p* is the number of parameters, *Y* is a vector of outcomes, *X* is a *N* x *p* matrix of covariates, and *β* is a vector of effects. The size of the penalty is determined by *λ*, which can be found via cross-validation to minimize prediction error. Alternatively, one can choose a stricter threshold for *λ* at 1 standard error above the minimum prediction error (to conservatively allow for error in the estimate of the optimal *λ*) [12]. Although the lasso can find a solution under multicollinearity, if a group of highly correlated variables is present the lasso tends to arbitrarily select one variable and set the others to zero [14].

##### Elastic net

The elastic net was proposed to overcome some of the limitations of the lasso method. It uses a combination of the L1 lasso penalty and the L2 ridge penalty:

2 $$ \underset{\beta }{\min}\left\{\frac{1}{2N}{\sum}_{i=1}^N{\left({y}_i-{\sum}_{j=1}^p{x}_{ij}{\beta}_j\right)}^2+\lambda \left(\frac{1}{2}\left(1-\alpha \right){\sum}_{j=1}^p{\beta}_j^2+\alpha {\sum}_{j=1}^p\left|{\beta}_j\right|\right)\right\}, $$

where *0 ≤ α ≤ 1,* and other parameters are defined as above in (1). The choice of α determines whether the penalty is closer to a ridge penalty (α = 0) or a lasso penalty (α = 1). The choice of both α and λ can be determined via cross validation [14]. Unlike the lasso, when predictors are collinear, the elastic net tends to classify groups of highly correlated variables as all either zero or nonzero. In many cases the elastic net provides better performance than the lasso [14].

##### Sparse models with clustering

Hierarchical clustering is a way of grouping variables with similar behavior across observations. Agglomerative clusters are built from the bottom-up by joining the “closest” clusters at each step according to defined distance and linkage functions, and the distance becomes the “height” at which clusters are joined. For the census data we propose to define distance as one minus the absolute value of the Spearman correlation coefficient. Complete linkage is a useful choice here as it maintains the original scale of the distance measures (in this case, from 0 to 1), and the height is therefore interpretable. The resulting clusters are represented via a dendrogram (see Additional file 1) [15].

Cluster membership can be defined by cutting the dendrogram at a specific height, so that any observations that are joined at a height lower than that value are members of a cluster. A more objective method is to identify statistically significant clusters via a bootstrap [16]. This method resamples participants to identify which clusters of variables often appear, measuring stability. Note that with either method, many clusters may contain only one variable. If the number of clusters is small relative to *n*, clustered variables can be summarized into a single measure, and models can then be fit using multiple regression models. However, if a large number of clusters are present, a better choice is to use cluster membership to fit group lasso or sparse group lasso models. The sparse group lasso is particularly useful, as it has penalties at both the group and individual level, allowing for sparsity across and within groups [17–19].

#### Tree ensemble methods

Another group of popular machine learning methods are based on tree ensembles, where many decision trees are fit to the data. Decision trees rely on recursive binary partitioning, that is, at each step (node) in the tree, the observations are split into two daughter nodes depending on some function of the predictor variables. Often, methods aggregating many trees (ensembles) outperform single tree based methods [20].

##### Random forests

The random forests method is useful for high-dimensional data. Underlying the method are many Classification and Regression Trees run on bootstrap samples of the dataset [15]. The relative impact of each variable on prediction accuracy is characterized using the variable importance score (VIMP), calculated by permuting each variable and re-fitting the random forest, and assessing how this impacts accuracy. VIMP scores provide a way of ranking predictors relative to each other, but choosing a threshold for the VIMP is often done post-hoc.

Recently, Ishwaran and Lu (2019) [21] proposed a resampling based calculation of the standard errors for the VIMP. We propose to use this standard error to inform variable selection via the confidence interval. Based on the estimated VIMP scores and their standard errors, we can create 100*(1-*α/p*)% confidence intervals for each variable. If the confidence interval excludes zero, we can conclude that there is evidence that the variable improves prediction, and therefore infer that there is a relationship between that variable and the outcome of interest.

##### Bagging

Bagging was a predecessor to random forests, and can be thought of as a special case. In the standard interpretation, at each node a random subset of variables (often choses to be *p*/3) are evaluated as candidates for splitting. In bagging, all *p* variables are considered for possible splitting. This tends to yield a smaller subset of variables with high VIMP scores, which may be better for our purposes of identifying the best variables [20]. We note that, as this is a special case of random forests, we can use the same resampling-based approach to define confidence intervals for the VIMP scores and accomplish variable selection.

##### Bayesian Additive Regression Trees (BART)

Like Random Forests and Bagging, BART is a tree ensemble method; however, it builds a set of trees using repeated draws from a Bayesian probability model. Similar to the VIMP of random forests, the relative importance of a given variable can be characterized using the variable inclusion proportion, defined as the number of splitting rules based on the variable out of the total number of splits. We can obtain an empirical estimate of the null distribution for the variable inclusion proportions by permuting the outcomes and re-fitting the BART algorithm. After these are obtained, three thresholds for variable selection have been proposed. The first, the local threshold, uses the null distribution of each individual variable, and if the fitted inclusion proportion is greater than its 1-α quantile under the null, that variable is selected. A more restrictive criterion (Global SE) increases the threshold using the local mean and standard deviation with a global multiplier determined based on the permutation distribution of all variables. The most restrictive criterion (Global Max) requires that the inclusion proportion is greater than the 1-α quantile of the maximum inclusion proportions (across all variables) from each permutation.

### Simulation study

Machine learning methods are typically evaluated by their ability to predict outcomes. In this study, we are interested in a different question: how well does each method correctly identify the subset of census variables which are truly associated with the outcome of interest? Therefore, we conducted a simulation-based experiment, generating outcomes which have known associations with a small subset of census variables.

The data structure of the census variables is complex; measures may exhibit non-normal distributions, contain excess numbers of zeros, and some variables are highly collinear (see Additional file 2). To fully reflect this complexity, we used observed census variables for PA prostate cancer cases [3] as the basis of our simulation. For computational tractability, we randomly selected 1000 variables and 2000 individuals. Missing values were imputed using median substitution, and all variables were standardized (mean = 0, standard deviation = 1). Let ***X*** be the data matrix corresponding to the full set of 1000 neighborhood variables. We define ***X***_***T***_ as the matrix with columns representing variables truly associated with the outcome of interest, *Y*. The full set of predictors, *p*, also contains many other predictors*.* We define matrix ***X***_***0***_ to be the matrix of columns not directly related to *Y*. The variables in ***X***_***0***_ which are highly correlated with variables in ***X***_***T***_ are considered “surrogate” variables.

**Fig. 1:**
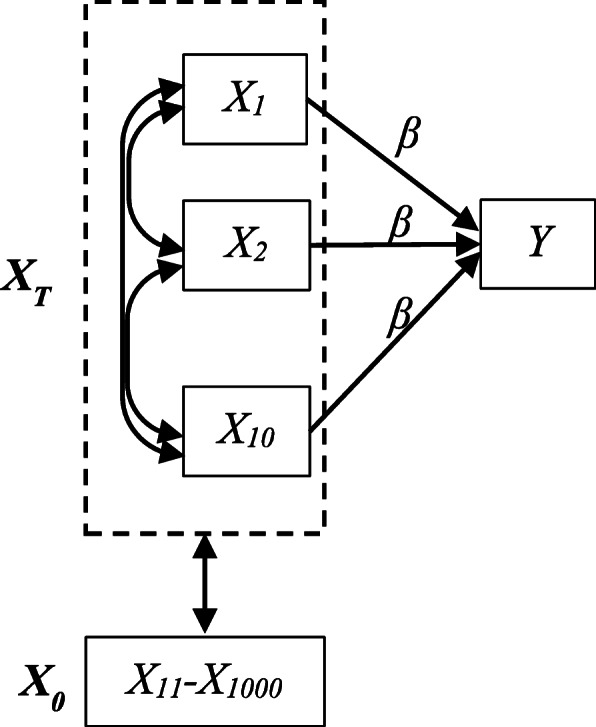
Simulation model

We selected 10 variables to be members of ***X***_***T***_, where 5 variables exhibited marked collinearity (*X*_*1*_*-X*_*5*_, correlation > 0.95 with at least 1 other variable), and 5 variables which exhibited modest or low collinearity (*X*_*6*_*-X*_*10*_, correlation < 0.6 with all other variables). Outcomes were simulated according to the structure shown in Fig. 1. We considered both binary and continuous outcomes as they are commonly seen in medical research with the mean models logit(*E*(*Y*)) = *β* ′ ***X***_***T***_ for binary *Y* and *E*(*Y*) = *β* ′ ***X***_***T***_ for continuous *Y*, with errors distributed as *N(0,1)*. Effect size (*β*) was equal for each member of ***X***_***T***_, with *β* = 0.22 for binary outcomes and *β* = 0.11 for continuous outcomes. The size of *β* was set as the effect size in a single univariable test for which we would have at least 80% power with 5*10^− 5^ 2-sided type-I error to detect the effect when *N* = 2000.

We simulated outcomes (*Y*) 500 times. In practice, variable selections are often internally validated by withholding a portion of the dataset. Therefore, we randomly assigned 2/3 of the data to be the discovery set and the remaining 1/3 to be the validation set. The algorithms discussed above were applied to each set of simulated outcomes. Candidate variables selected in the discovery set were then validated in the withheld 1/3 sample, using a series of univariable regression models, considering any variable with a P-val < 0.05 to be validated, similar to the approach taken in some GWAS studies [22]. For the random forest method, which often identified a large number of variables in the discovery set, we also explored using a multivariable lasso model in the validation in an attempt to reduce potential confounding in the validation step. Table 1 lists each method tested, along with the selection rule in the discovery set. All models were fit using R software (version 3.5) [23]. Software used to fit models and run simulations is available on github. (https://github.com/BethHandorf/neighborhood-machine-learning).

**Table 1:** Candidate methods

Abbreviation	Description	Selection rule	R packages
UNIV-BFN	Univariable models with Bonferroni-adjusted p-val	*P* < 5*10^−5^	base R [23]
LASSO-MIN	Lasso with λ chosen at the minimum prediction error	β ≠ 0	glmnet [24]
LASSO-1SE	Lasso with λ chosen at 1 SE above the minimum error	β ≠ 0	glmnet
ELNET-MIN	Elastic net, grid search for α (0.05–0.95 by 0.05), λ at min	β ≠ 0	glmnet
ELNET-1SE	Elastic net, grid search α (0.05–0.95 by 0.05), λ at 1 SE	β ≠ 0	glmnet
HCLST-CORR-SGL	Hierarchical clustering, groups with corr > 0.8, sparse group lasso	β ≠ 0	SGL [25]
HCLST-BOOT-SGL	Hierarchical clustering, groups from bootstrap, sparse group lasso	β ≠ 0	SGL, pvclust [16]
RF	Random Forests algorithm with bootstrap-based confidence intervals for the variable importance scores	99.995% CI > 0	randomForestSRC [26]
BAGGING	Similar to Random Forests, but with all variables considered candidates for splitting at each node	99.995% CI > 0	randomForestSRC
BART-LOCAL	Bayesian Additive Regression Trees, local criteria for Inclusion Proportion (IP)	IP > 0.95 quantile of local distribution	bartMachine [27]
BART-GLOBALSE	Bayesian Additive Regression Trees, global SE criteria for IP	IP > threshold from local distribution with global multiplier	bartMachine
BART-GLOBALMAX	Bayesian Additive Regression Trees, global Max criteria for IP	IP > 0.95 quantile of global max distribution	bartMachine

#### Comparison of methods: performance assessment

Performance was quantified by which methods identified a large proportion of true positive variables (***X***_***T***_) and minimized false positive variables (***X***_***0***_). We also considered more flexible success metrics where true positives were considered as the identification of either a target variable or a good surrogate (correlation > 0.8 with target), and false positives were considered as those not a target variable or a surrogate. The threshold of 0.8 was chosen as it is a commonly used value for determining suitability of surrogate endpoints in clinical studies [28, 29]. We also considered a composite metric, the F2 score. This is a measure of accuracy combining the Positive Predictive Value (PPV, sometimes termed precision) and the sensitivity (sometimes termed recall). The F2 score is a specific case of the general F score, which gives greater weight to the sensitivity [30].

$$ F2=\frac{\left({2}^2+1\right)\ast TP}{\left({2}^2+1\right)\ast TP+{2}^2\ast FN+ FP\ } $$

Where TP is the number of true positives, FN is the number of false negatives, and FP is the number of false positives. When comparing models, a F2 score closer to 1 denotes the preferred model. We chose the weighted F2 as we believe that in this application, priority should be given to the ability to detect more true positive variables. Finally, we determined the average detection rate for the individual variables, and evaluated how effect size estimates from univariable models were related to the likelihood of detection.

### Application to PA prostate cancer cases

To illustrate these methods in practice, we applied the most promising algorithms (those with the most true positives and fewest false positives) from the simulation study to the full dataset used in the prior NWAS study, which linked prostate cases from a PA Department of Health registry to social-environmental variables obtained from the US Census [3] The binary outcome of interest was aggressive (high stage AND grade) prostate cancer [3]. This cohort of white prostate cancer patients diagnosed between 1995 and 2005 contained 76,186 individuals (8% with aggressive disease). There were 14,663 census variables evaluated for association with the outcome. We included census variables as predictors, along with age and year of diagnosis. The data was split into discovery (2/3) and validation (1/3) samples. As above, variables selected in the discovery set were tested using univariable regression in the validation set, using a *p*-value cutoff of 0.05. We then compared which variables were selected by the most promising methods (from the simulation study) in the full study population to those found by the original NWAS method.

## Results

### Comparison of methods

Table 2 shows the mean number of variables detected by each method, broken down into true positives and false positives. The strict definition considers true positives to be identification of variables in ***X***_***T***_, while the relaxed definition allows for surrogate variables. For the false positives, the strict definition shows the number of members of ***X***_***0***_ which were identified, while the relaxed definition shows the number of selected members of ***X***_***0***_ which did not have a substantial correlation with an element of ***X***_***T***_. The number of false positives was substantially reduced under the relaxed definition, especially for methods which identify groups of correlated predictors (e.g. elastic net, sparse group lasso), demonstrating that many of the “false positive” results were identified due to their relationship with a “true positive” variable.

**Table 2:** Simulation study results

A. Binary outcome	Strict			Relaxed^a^		
	TP (N/10)	FP (N/990)	F2	TP (N/10)	FP (N/953)	F2
UNIV-BFN	4.09	32.49	0.267	5.13	12.70	0.443
LASSO-1SE	3.84	6.05	0.383	5.53	3.71	0.559
LASSO-MIN	4.25	9.01	0.399	5.98	6.53	0.569
ELNET-1SE	5.26	20.51	0.405	6.21	9.33	0.560
ELNET-MIN	5.53	26.11	0.393	6.61	14.40	0.548
HCLST-CORR-SGL	**5.40**	**17.91**	**0.428**	**6.39**	**7.09**	**0.597**
HCLST-BOOT-SGL	5.20	16.66	0.420	6.35	7.07	0.594
RF	3.53	18.41	0.281	4.91	7.68	0.462
BAGGING	3.56	13.94	0.308	4.73	6.70	0.456
BART-LOCAL	4.68	15.66	0.387	6.32	7.13	0.591
BART-GLOBALSE	1.96	0.53	0.228	2.24	0.22	0.261
BART-GLOBALMAX	0.01	0.00	0.001	0.01	0.00	0.001
B. Continuous outcome	Strict			Relaxed^a^		
	TP (N/10)	FP (N/990)	F2	TP (N/10)	FP (N/953)	F2
UNIV-BFN	4.83	39.57	0.286	5.90	17.47	0.468
LASSO-1SE	2.88	4.49	0.298	4.33	2.42	0.454
LASSO-MIN	4.61	10.52	0.419	6.47	7.60	0.599
ELNET-1SE	3.88	8.27	0.366	4.87	3.29	0.500
ELNET-MIN	5.18	14.79	0.433	6.61	8.88	0.598
HCLST-CORR-SGL	**5.82**	**19.86**	**0.444**	**6.81**	**8.51**	**0.617**
HCLST-BOOT-SGL	5.52	17.03	0.441	6.72	8.45	0.610
RF	4.63	28.46	0.316	5.93	14.26	0.493
BAGGING	4.40	25.73	0.314	5.81	12.96	0.494
BART-LOCAL	5.14	18.35	0.404	**6.80**	**8.34**	**0.617**
BART-GLOBALSE	2.40	0.93	0.274	2.85	0.41	0.326
BART-GLOBALMAX	0.01	0.00	0.002	0.01	0.00	0.002

*TP* True positive, *FP* False positive; boldface denotes best performing method by F2 statistic

^a^Under the relaxed definition, if a true variable or its surrogate was selected, that variable was considered to be identified. Surrogates are therefore no longer in the pool of potential false positives, but the maximum number of true positive variables remains 10

For binary outcomes, the sparse regression method identifying the fewest false positive results was the lasso with the restrictive 1SE penalty (LASSO-1SE), while elastic net with the less restrictive penalty (ELNET-MIN) identified the largest number of true positives. When considering the sparse group lasso, the simpler correlation-based threshold to define clusters worked somewhat better than the more complex bootstrap-based cluster detection (although results were largely similar). The correlation-based clustering generated more clusters on average than bootstrap-based cluster selection (837 vs 772), so more restrictive cluster definitions may have led to these differences. Of the tree ensemble methods, BART-LOCAL performed the best. It substantially outperformed both RF or BAGGING. BART-GLOBALSE and BART-GLOBALMAX were too restrictive, identifying very few true positive variables.

Comparing the methods, there was generally a trade-off between the number of true positives and false positives. However, certain strategies were clearly inferior to others (dominated), with higher false positive rates and lower true positive rates than other methods. Univariable models and the random forests based models can be eliminated from consideration in future studies based on this criterion. When assessing the combined F2 measure of performance, many of the penalized models performed particularly well, with HCLST-CORR-SGL doing the best overall. BART-LOCAL also did well, particularly under the relaxed definition. The F2 measure indicates that these methods have particularly good sensitivity, while also considering their PPV.

The results were largely similar for both continuous (Normal) and binary outcomes. We did find that Random Forests (RF) identified more variables (both false positives and false negatives) for the continuous outcome, while elastic net identified more variables with the binary outcome; however, the same method (HCLST-CORR-SGL) had the highest F2 statistics for the strict and relaxed definitions. For the continuous outcome, under the relaxed definition, BART-LOCAL did as well as HCLST-CORR-SGL.

Considering the individual variables, those chosen from areas of high correlation (*X*_*1*_*-X*_*5*_) were selected less often than those from areas of moderate to low correlation (*X*_*6*_*-X*_*10*_), and there was more variability in detection rates for *X*_*1*_*-X*_*5*_. (See Additional file 3) Unsurprisingly, the lasso had notably low detection rates for *X*_*1*_*-X*_*5*_.

### Performance assessment: exploration of false negatives/impact of correlated data

One unexpected finding was the very low true positive rate for certain variables. For the Bonferroni-adjusted univariable analyses, we would expect each variable to be detected in 39–40% of simulations, based on power to detect effects in training and validation sets. However, the proportion of time a variable was chosen (binary case) ranged from 0 to 97% (see Table 3). These results were attributable to confounding within ***X***_***T***_. Confounding of the relationship between a predictor *X* and an outcome *Y* occurs when a third factor is associated with both *X* and *Y*. Here, there were small to moderate correlations between the members of ***X***_***T***_ **(**see Additional file 4). Therefore, when variables were analyzed separately, the regression model was misspecified due to confounding. As shown in Table 3, the estimated effects from univariable models (i.e. our UNIV-BFN models) relate to the proportion of times a variable is identified. Variables with estimated effects > 0.22 (larger than the truth) were more likely to be selected, while variables with estimated effects < 0.22 (smaller than the truth; X_4_, X_5_, X_10_) were almost never identified. Unfortunately, even methods like the lasso which simultaneously consider all variables did not provide substantial improvements in detection rates of rarely selected variables, nor did using a multivariable (lasso) model in the validation step in place of the univariable regressions.

**Table 3:** Effect of confounding on detection rate (binary outcome)

	Mean β (true = 0.22)	Detection rate (UNIV-BFN)	Detection rate (HCLST-CORR-SGL)	Detection rate RF with lasso validation
X_1_	0.223	0.25	0.586	0.244
X_2_	0.343	0.972	0.778	0.126
X_3_	0.252	0.546	0.372	0.132
X_4_	0.152	0.022	0.014	0.028
X_5_	0.085	0	0	0.01
X_6_	0.233	0.316	0.800	0.662
X_7_	0.281	0.812	0.928	0.71
X_8_	0.228	0.388	0.768	0.382
X_9_	0.280	0.786	0.928	0.728
X_10_	0.032	0	0.024	0.006

### Application to PA prostate cancer cases

We assessed associations between the census variables and the outcome of aggressive Prostate Cancer (PCa) using HCLST-CORR-SGL, the best-performing method for binary outcomes. After applying the hierarchical clustering algorithm with a threshold of 0.8, 10,888 of the 14,663 variables were grouped with at least one other variable. Of the 6865 clusters identified, 3090 had two or more variables (max 244), and 3765 clusters contained only one variable. HCLST-CORR-SGL identified 19 census variables in 13 clusters as predictors of aggressive disease (Additional file 5), as well as year of diagnosis and patient age. One variable found in the NWAS study was identified by this approach, and two variables/clusters in the NWAS were highly related to variables identified by HCLST-CORR-SGL. These overlapping variables are described in Table 4. We note that the results from HCLST-CORR-SGL were sparse within clusters; nevertheless, the algorithm does not force selection of a single representative variable from each cluster. Therefore, some highly related variables were chosen (e.g. PCT_SF3_PCT065I007 and PCT_SF3_PCT065A007).

**Table 4:** Results from full data: Variables identified as associated with PCa aggressiveness by both HCLST-CORR-SGL and NWAS

SGL variable(s)	Domain: Description	NWAS variable	Correlation(s)
PCT_SF3_PCT050102	Poverty: % Ratio of Income to Poverty level for persons aged 45–54 under 0.50	PCT_SF3_PCT050102	1.0
PCT_SF3_HCT005092	Housing/Income: %Renter-occupied housing units built 1939–1949 with householder aged 25–34	PCT_SF3_HCT015042	0.912
PCT_SF3_PCT065I007	Employment/Transportation: % White Only (non-Hispanic) Worker taking public transportation (trolley or streetcar) to work	PCT_SF3_P030007	0.935
PCT_SF3_PCT065A007	% White Only Worker taking public transportation (trolley or streetcar) to work	PCT_SF3_P030007	0.935

## Conclusions

In simulation studies, we found that methods using hierarchical clustering combined with sparse group lasso (HCLST-CORR-SGL) performed the best at identifying variables with true associations (or their surrogates), while providing control of false positive results. This conclusion is based on the method’s F2 scores in simulated data, a combined measure of accuracy which gives greater weight to the method’s sensitivity. HCLST-CORR-SGL used clustering to directly address the complex correlation structure of the data, which may have led to improvements in the ability of penalized regression to detect true positive variables. We showed that the simpler threshold-based approach was sufficient to define meaningful clusters. However, none of the approaches we assessed solved the issue of low detection rates for the variables subject to confounding towards the null, even though the sparse regressions are based on multivariable models.

We applied the HCLST-CORR-SGL to our full dataset and compared findings from these machine learning methods to our previously published NWAS, under the assumption that variables that replicated across methods were more likely to represent true findings. We note that in our simulation study, outcomes were generated completely independently, while the observed outcomes in the full dataset likely had spatial effects which were not accounted for in the machine learning approaches applied here. Nevertheless, HCLST-CORR-SGL did independently replicate three out of 17 NWAS variables (i.e. the identification of the same or a highly correlated variable) which did take into account potential spatial effects.

Previous studies of social-environmental factors often selected census variables a priori. These studies showed that single variables representing single domains (e.g. % living below poverty) were associated with advanced prostate cancer and cancer more broadly [4]. Interestingly, the NWAS and machine learners consistently identified more complex variables that combined domains related to race, age, and poverty with household or renter status. Thus, findings from these empirical methods could serve to be hypothesis-generating, suggesting interactions among domains that are often considered individually in current social-environmental studies. For example, the top hit from the previous NWAS (PCT_SF3_P030007) had a correlation of 0.93 with two variables identified by HCLST-CORR-SGL (PCT_SF3_PCT065I007 and PCT_SF3_PCT065A007). All three are markers of employment and transportation, a combination of two different domains.

This study has several limitations. First, although we assessed several popular machine-learning algorithms for variable selection, there are many other approaches. We considered principal components regression, as it is commonly used with highly collinear data, but ultimately did not include it because the results were difficult to interpret and required arbitrary thresholds. Other popular machine learning approaches that use a “black box” algorithm for prediction (e.g. neural networks) were not readily useable for variable selection and therefore were not included. Most Support Vector Machines (SVM) based methods are not readily used for variable selection; we considered a sparse SVM method, but found that it was computationally infeasible to implement [31]. We also did not evaluate predictive accuracy of the various methods, as it was not of primary interest. Further, we intentionally designed a situation where variables had small effects compared to the random error, [3] so even a perfect method would have relatively low predictive ability. In real-world cases, the social-environmental variables would be combined with patient-level variables, giving the models much better predictive accuracy; we did not do so here to isolate the effect of method choice on selection of neighborhood predictors. Also, this work specifically considered the use of US Census variables; if researchers will use other sources of social-environmental data which is appreciably different in structure, it would be prudent to evaluate some of the better performing methods via simulation. In such a case, researchers could use our simulation methodology and code as a template to help guide their choice of model. Finally, for computational tractability, the size of the dataset used in simulations was limited to 1000 variables and 2000 subjects, much smaller than the full dataset upon which this study is based. In future studies, we will assess the impact of spatial relationships and the rate of true associations on the method’s performance. We will also consider cases where the data-generating model is non-linear, includes interactions, and uses patient-level predictors. Other directions needing further study include evaluating and developing methods for separate sources of social and environmental data (e.g. measures of exposure to pollution), and determining whether such predictors should be analyzed separately, or combined in a unified framework. Our work also demonstrates the need for new methods with improved capacity for variable selection in the presence of confounding.

In this era of Big Data and Precision Medicine, [32, 33] the importance of neighborhood and other environmental data will continue to grow. Given the complex structure and high dimensionality of environmental data, researchers should continue to develop machine learning approaches for this area. For complex diseases like cancer, the analysis of multilevel, mixed feature datasets (including environmental, biological, and behavioral features) will likely be needed to inform health disparities, disease prevention and clinical care, motivating the development of new analytical approaches.

## Supplementary Information

**Additional file 1.** Dendrogram for correlation between variables. Dendrogram showing the relationships between the 1000 elements of the covariate matrix X. The horizontal red line represents a correlation of 0.8.

**Additional file 2. **Distribution of 10 variables associated with simulated outcomes. Histograms showing the distributions of *X*_*1*_*-X*_*10*_, the elements of *X* used to simulate the outcomes Y.

**Additional file 3.** Detection rates for each variable. Table showing the proportion of simulations where each varaible was selected (by each method).

**Additional file 4. **Correlation structure of 10 variables. Correlations structure of *X*_*1*_*-X*_*10*_, the elements of X used to simulate the outcomes Y. Blue represents a positive correlation and red a negative correlation, with darker colors indicating a stronger relationship.

**Additional file 5.** Full data results. Table containing all finidings when the HCLST-CORR-SGL method was applied to the full PA PCa dataset (binary outcome: diagnosis with aggressive PCa).

## Data Availability

Software and supplementary files available at https://github.com/BethHandorf/neighborhood-machine-learning . The dataset(s) supporting cannot be made publicly available by the authors for ethical and legal reasons. They include geocoded data at the census tract level linked to individual, anonymized records and releasing PA registry data is against the data use agreement. The U.S. Census data used for this analysis can be downloaded from www.socialexplorer.com . Researchers can obtain data on cancer cases directly from the Pennsylvania Cancer Registry, upon request to Wendy Aldinger, RHIA, CTR -- Registry Manager 1–800–272-1850, ext. 1, wealdinger@pa.gov, https://www.health.pa.gov/topics/Reporting-Registries/Cancer-Registry/Pages/Cancer%20Registry.aspx .

## Abbreviations

BART: Bayesian Additive Regression Trees
BNF: Bonferroni
CORR: correlation
ELNET: Elastic Net
FN: False Negative
FP: False Positive
GWAS: Genome-Wide Association Studies
HCLST: Hierarchical Clustering
LASSO: Least Absolute Shrinkage and Selection Operator
MAX: Maximum
MIN: Minimum
NWAS: Neighborhood-Wide Association Study
PA: Pennsylvania
PCa: Prostate Cancer
PPV: Positive Predictive Value
RF: Random Forests
SE: Standard Error
SGL: Sparse Group Lasso
SVM: Support Vector Machines
TP: True Positive
US: United States
UNIV: Univariable
VIMP: Variable Importance Score
