# Supplementary figures and images for: Variable selection in social-environmental data: sparse regression and tree ensemble machine learning approaches
Source: BMC Med Res Methodol. 2020 Dec 10;20:302. doi: 10.1186/s12874-020-01183-9 (PMC7727197; doi:10.1186/s12874-020-01183-9)

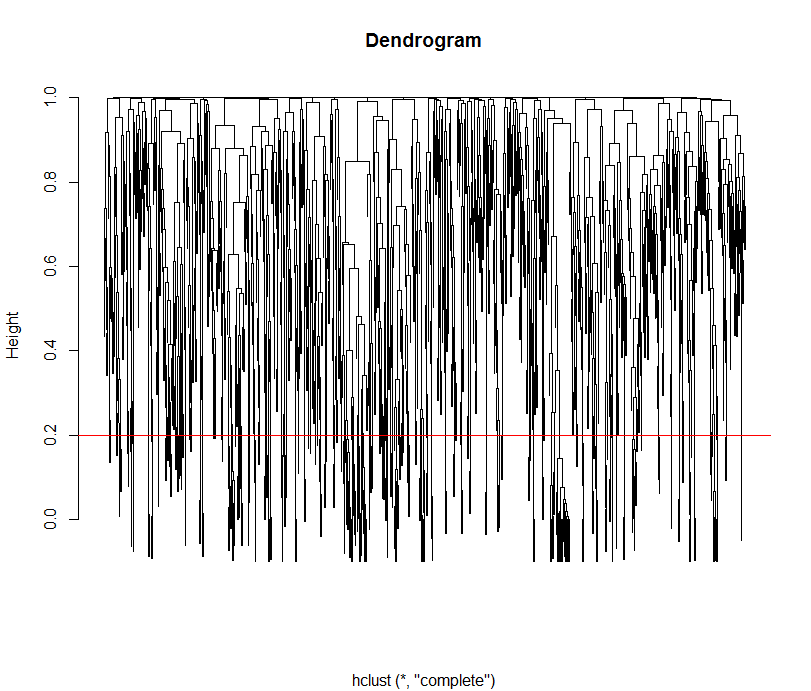

Supplement: Supplementary file 1 — Additional file 1. Dendrogram for correlation between variables. Dendrogram showing the relationships between the 1000 elements of the covariate matrix X. The horizontal red line represents a correlation of 0.8. [file 12874_2020_1183_MOESM1_ESM.tiff]

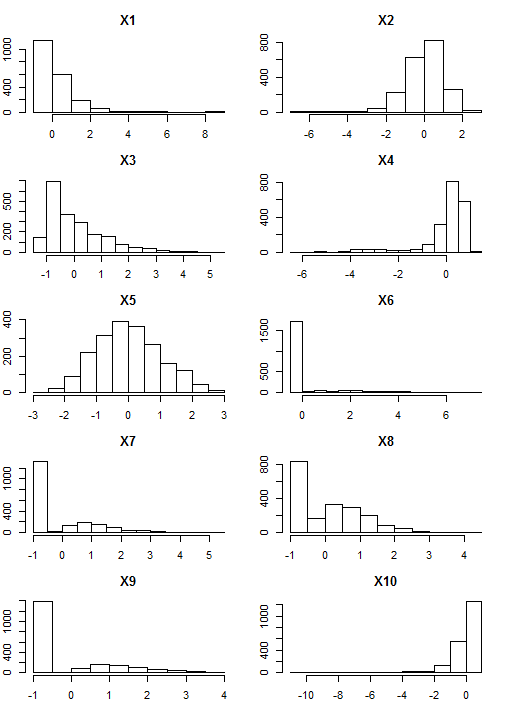

Supplement: Supplementary file 2 — Additional file 2. Distribution of 10 variables associated with simulated outcomes. Histograms showing the distributions of X1-X10, the elements of X used to simulate the outcomes Y. [file 12874_2020_1183_MOESM2_ESM.tiff]

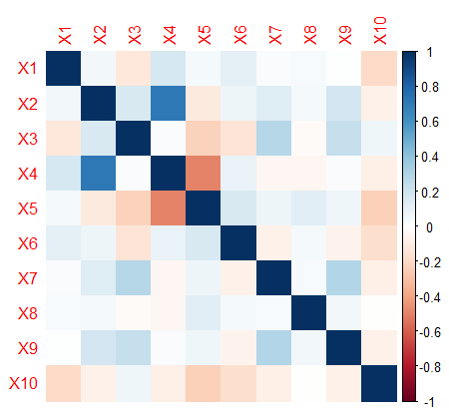

Supplement: Supplementary file 4 — Additional file 4. Correlation structure of 10 variables. Correlations structure of X1-X10, the elements of X used to simulate the outcomes Y. Blue represents a positive correlation and red a negative correlation, with darker colors indicating a stronger relationship. [file 12874_2020_1183_MOESM4_ESM.tif]
